# Editorial: Research in social psychology, prevention activities and mental health promotion

**DOI:** 10.3389/fpsyg.2024.1373715

**Published:** 2024-02-23

**Authors:** Brais Ruibal-Lista, José Enrique Moral-García, Alba González-Palomares, Manuel Loureiro, Sergio López-García

**Affiliations:** ^1^EUM Fray Luis de León, Catholic University of Ávila, Valladolid, Spain; ^2^Research Group on Physical Activity, Sports and Health (GIADES), Pontifical University of Salamanca, Salamanca, Spain; ^3^Faculty of Education, University of Jaén, Jaén, Spain; ^4^Faculty of Education, University of Salamanca, Salamanca, Spain; ^5^Faculty of Social and Human Sciences, University of Beira Interior, Covilhã, Portugal; ^6^Research Center in Sports Sciences, Health Sciences and Human Development (CIDESD), Vila Real, Portugal; ^7^Faculty of Education, Pontifical University of Salamanca, Salamanca, Spain

**Keywords:** editorial, research, exercise, social promotion, prevention activities and mental health promotion

As guest editors for Frontiers in Psychology, we are honored to present the outcomes of the Research Topic entitled “*Research in social psychology, prevention activities, and mental health promotion.”* This scientific compendium is designed to underscore the significance of regular physical activity in combating sedentary lifestyles and promoting overall health.

This Research Topic not only seeks to emphasize the importance of physical activity in mental health but also addresses the necessity of social policies promoting healthy habits from various perspectives: preventive, educational, economic, and social. It emphasizes that these policies should not be seen as a cost but rather as a crucial investment in the health and quality of life of society as a whole.

Researchers and educators were invited to contribute innovative research in the field of healthy habits, physical activity, and social psychology. The goal was to publish rigorous scientific articles that provide new insights into promoting healthy lifestyles and physical fitness, while also advocating for preventive measures and improving the quality of life across all age groups.

The Research Topic has achieved remarkable figures; shortly after closing, 16 articles involving 55 authors have been published, accumulating over 23,000 reads and around 4,000 full articles downloads. Finally, it is important to highlight the demand for the quality of the publications. In total, 73 manuscripts were submitted and 16 were accepted, so the publication rate is around 22%.

Due to the substantial number of articles, we have decided to categorize their conclusions into three sections:

## Section 1 - Studies on social behaviors, isolation, depression, and suicide

Ikenouchi et al.'s “*Effect of the personality traits of healthy Japanese workers on depressive symptoms and social adaptation, and on the achievement rate of exercise therapy to prevent major depression”:* these authors demonstrated that depressive symptoms and social adaptation were differently associated with personality traits and success rates before and after exercise therapy.

Xing et al.'s “*Exercise adherence and suicidal ideation of Chinese college students: a chain mediation model test”:* among other findings, they showed that physical exercise can directly predict suicidal ideation in Chinese college students.

Wang et al.'s “*From experience to expectation: The reverse effect of power on purchasing impulsiveness”:* this research presents a new theoretical perspective on the relationship between power and purchasing impulsivity, proposing a power experience-expectation model that suggests consumer purchasing impulsivity is influenced by both experience and power expectations.

Wu et al.'s “*Consistency in personality trait judgments across online chatting and offline conversation”:* participants consistently judged individuals in terms of empathy and the Big Five personality traits in both online chat and offline conversation contexts. These findings contribute to understanding how people are perceived across different environments.

Yamaguchi et al.'s “*The influence of vulnerability on depression among Japanese university athletes”:* this study observed that appropriate psychological support in athletes can reduce depression and improve mental health, especially in those with higher vulnerability levels.

Yi and Li “*The influence of local government competition on residents' perceptions of social fairness—Evidence from China*”: results indicate that such competition broadens the perception of social injustice, influencing through income disparity, reduction of public goods, and an increase in corruption. These findings have practical implications for promoting common prosperity and strengthening the capacity of local government.

## Section 2 - Social relationships among youth, social relationships in the elderly, and compulsive internet and smartphone use

Parti “*What is a capable guardian to older fraud victims? Comparison of younger and older victims' characteristics of online fraud utilizing routine activity theory”:* it was observed that computer usage time influences young people's vulnerability to online fraud. However, older individuals are less likely to employ technical protections, such as covering cameras, monitoring identity theft, and freezing credit cards, and are less likely to report scams.

Zhao and Zheng “*The effect of peer victimization on adolescents' revenge: the roles of hostility attribution bias and rumination tendency”:* results suggest that peer victimization is positively related to revenge, with this relationship being stronger in individuals with a tendency toward concrete experiential rumination than in those with abstract analytical tendencies.

Liu et al.'s “*Effect of physical exercise on social adaptability of college students: Chain intermediary effect of social-emotional competency and self-esteem”*: the authors observed that physical exercise not only directly affects college students' social adaptation but also has an indirect effect through the independent mediating role of social-emotional competence and self-esteem.

Huang et al.'s “*The relationship between sense of community and general well-being of Chinese older adults: A moderated mediation model”:* findings suggest that fostering community participation can enhance the overall wellbeing of older adults, contributing to building stronger societies in Chinese cities.

Wanqing et al.'s “*Smartphone addiction and cross-cultural adjustment among overseas Chinese students: The role of emotion regulation beliefs and strategies”:* this study demonstrates the correlation between emotion regulation beliefs regarding smartphone addiction and cross-cultural adaptation, as well as the detrimental effects of emotional neglect in childhood; these components should be further addressed in future studies.

Du and Zhang “*Analysis of the mediating effects of self-efficacy and self-control between physical activity and Internet addiction among Chinese college students”:* it was demonstrated that physical activity not only has a direct negative correlation with Internet addiction but also influences through two indirect forms: the mediating role of self-control and the chain mediating role of self-efficacy and self-control.

Iraurgi et al.'s “*Adaptation to Spanish of the “Relational Needs Satisfaction Scale”: Translation and psychometric testing”:* the authors validated the Spanish version of the RNSS and demonstrated that it is a valid and reliable measure of the intended construct.

## Section 3 - Effects of physical exercise on health and sports performance

Fujino et al.'s “*Decreased step count prior to the first visit for MDD treatment: a retrospective, observational, longitudinal cohort study of continuously measured walking activity obtained from smartphones”:* it was found that a significant decrease in daily step count preceded the formal diagnosis of Major Depressive Disorder (MDD) by ~2 weeks, followed by a gradual increase after diagnosis and presumed treatment. These findings suggest the utility of objective and continuous measures to identify the development of MDD before it impacts work productivity.

Sánchez Cabaco et al.'s “*Mediation effects of cognitive, physical, and motivational reserves on cognitive performance in older people”:* the importance of measuring cognitive reserve as a variable for diagnosing neurodegenerative diseases was demonstrated, but it is also essential to consider physical status and activity, as well as motivational aspects.

Wada et al.'s “*Exploring the effects of COVID-19 on motorcycle riding patterns and its importance”:* these authors observed that individuals who spent their leisure time riding motorcycles attributed greater importance to personal space and shared time with others. This suggests that such activity provided a means of practicing social distancing while sharing moments with peers, mitigating loneliness and isolation during the pandemic.

This Research Topic, through its diverse contributions, provides a comprehensive insight into the intersection of physical activity, social psychology, and mental health promotion.

The conclusions drawn from the studies highlight the importance of physical activity and social contact in various aspects of life, ranging from preventing mental disorders to improving social adaptability and overall wellbeing.

## Author contributions

BR-L: Writing – original draft. JM-G: Writing – original draft. AG-P: Writing – original draft. ML: Writing – original draft. SL-G: Writing – review & editing.

